# Entrepreneurs, Chance, and the Deterministic Concentration of Wealth

**DOI:** 10.1371/journal.pone.0020728

**Published:** 2011-07-21

**Authors:** Joseph E. Fargione, Clarence Lehman, Stephen Polasky

**Affiliations:** 1 Department of Applied Economics, University of Minnesota, St. Paul, Minnesota, United States of America; 2 College of Biological Sciences, University of Minnesota, St. Paul, Minnesota, United States of America; Erasmus University Rotterdam, The Netherlands

## Abstract

In many economies, wealth is strikingly concentrated. Entrepreneurs–individuals with ownership in for-profit enterprises–comprise a large portion of the wealthiest individuals, and their behavior may help explain patterns in the national distribution of wealth. Entrepreneurs are less diversified and more heavily invested in their own companies than is commonly assumed in economic models. We present an intentionally simplified individual-based model of wealth generation among entrepreneurs to assess the role of chance and determinism in the distribution of wealth. We demonstrate that chance alone, combined with the deterministic effects of compounding returns, can lead to unlimited concentration of wealth, such that the percentage of all wealth owned by a few entrepreneurs eventually approaches 100%. Specifically, concentration of wealth results when the rate of return on investment varies by entrepreneur and by time. This result is robust to inclusion of realities such as differing skill among entrepreneurs. The most likely overall growth rate of the economy decreases as businesses become less diverse, suggesting that high concentrations of wealth may adversely affect a country's economic growth. We show that a tax on large inherited fortunes, applied to a small portion of the most fortunate in the population, can efficiently arrest the concentration of wealth at intermediate levels.

## Introduction

The distribution of wealth is a fundamental property of how society is structured and has myriad economic, political, and social implications. The right to keep a large part of what one earns is one of the basic tenets of democratic capitalism, which provides incentives to invest and contribute to the productivity of the economy. However, large concentrations of wealth raise equity issues and may be incompatible with democracy itself; as put bluntly by U.S. Supreme Court Justice Louis Brandeis: “We can either have democracy in this country or we can have great wealth concentrated in the hands of a few, but we cannot have both.”

Models of the wealth distribution [1]–[5] have failed to capture the empirically observed large concentration of wealth in the top few percentiles, predicting too even a wealth distribution [6]. A range of explanations have been offered for this discrepancy. Some observers attribute the concentration of wealth to political factors [7] or to inherent properties of human nature such as differences in human capital (reviewed in [8]). In addition, even though empirical patterns show that savings rates increase with household earnings [9]–[11], many models of savings assume that individuals save to buffer against earnings shocks so that savings rates decline when an individual accumulates sufficient wealth [6], [12], [13].

Recent work has identified the importance of entrepreneurship in generating high concentrations of wealth [11], [14], [15]. Entrepreneurs differ in their investment strategies from those assumed by most economic models. Rather than being diversified (as assumed by Capital Asset Pricing Models, e.g. [16]), successful entrepreneurs often retain a majority of their wealth in ownership of businesses they lead (e.g. [17], [18]). There may be a variety of cultural reasons for this. For example, entrepreneurs may by nature be more confident in their ability to produce wealth through their own businesses than through the stock market, or may feel the need to retain ownership to signal confidence in their business or to retain decision making power, prestige, or other non-pecuniary benefits [17], [18]. Entrepreneurship is quite frequent–about 1 in 9 people in the United States is self-employed, and this rate of entrepreneurship has held steady over at least the past two decades [19].

## Analysis

We analyze whether a simple individual-based stochastic model that includes compounding returns can generate the highly concentrated wealth distribution observed among entrepreneurs in real populations. Before considering more complicated explanations, we believe it is useful to understand whether wealth concentration could occur due to the effects of chance alone. The effects of chance on wealth distribution may be revealed in models that track the wealth of individual entrepreneurs and include stochasticity, as opposed to more commonly used aggregate general equilibrium models, which do not allow for effects of stochastic variation among individuals. We isolate the role of chance by starting with assumptions that favor equality of wealth and exclude other factors that could lead to the concentration of wealth. We assume that all individuals have equal talent and begin with the same amount of capital. We also assume that business success in one year is not correlated with future business success. After exploring the implications of these assumptions, we test whether our conclusions are robust to variations in assumptions.

Among entrepreneurs, the dynamics of wealth concentration are determined largely by growth (or loss) of business worth. Therefore, we track capital wealth and assume that labor income does not factor into the growth of capital wealth. In economic terms, another way to arrive at this assumption is if all existing capital is invested, all capital income is reinvested, and consumption is equal to labor income. This allows us to track capital without the need to track labor income or consumption.

We assume all entrepreneurs begin with equal capital, set to 1 unit of wealth. In each time period (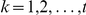) each entrepreneur (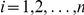) invests their capital and earns a return rate, 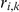, that is randomly drawn from a normal distribution with mean 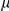 and variance 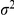. In reality, variance in return rate could be due to many factors, however the goal of our model is to assess the influence of chance alone. We investigate this by assuming all entrepreneurs are equal in all factors that could affect return rate, except for the effects of chance. Because returns are independent random draws for each individual for each period, in our simplest model we avoid (1) temporal autocorrelation, i.e., a successful entrepreneur in one period does not have an increased chance of getting a high rate of return in subsequent periods, (2) correlation across individuals in the same time period, i.e., all years have a constant average rate of return, and (3) differences among individuals in the chance of getting high or low returns. The number of individuals in this simplest model does not change, nor is there any explicit treatment of death. Thus, this version of the model describes the wealth of individual entrepreneurial families in a society where accumulated wealth is passed seamlessly to the next generation.

## Results

This simple model demonstrates that, with passing time, the proportion of wealth held by an arbitrarily small fraction of entrepreneurs asymptotically approaches 1–that is, a small proportion of entrepreneurs come to possess essentially all of the wealth. Given a rate of return 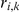, the factor by which entrepreneur 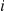
's capital increases in time period 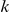 is 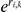. The total amount of capital accumulated by entrepreneur 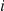 as of period 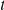 is 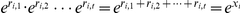, where 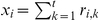. If the rates 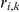 are drawn from a normal distribution with mean 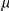 and variance 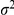, the exponential portion of this number (the sum of the rates) will be normally distributed with mean 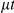 and variance 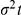. Then the total wealth is the individual cumulative wealth, 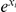, integrated over the probability density function of the normal distribution and multiplied by the number of individuals. The proportion of wealth held by an arbitrarily small percentage of the entrepreneurs is thus represented as the ratio of two integrals, with total wealth of the population in the denominator and wealth of the top percentage in the numerator.
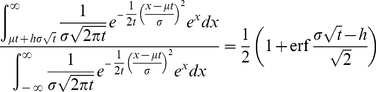
(1)


The integral in the numerator can represent the wealth in any segment of the population. Parameter 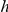 sets the lower limit of integration at a specific number of standard deviations above the mean. Because the integral extends to infinity, it captures the top portion of the wealth distribution , where 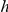 determines which top proportion is captured (e.g. 1%, 10%, etc.). When parameter 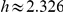, the integral starts at 99%, such that the numerator quantifies the wealth within the top 1% of the population. The notation “erf” refers to the “error function”, the sigmoidal function related to the cumulative normal probability distribution and defined by 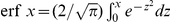. Since 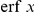 approaches 1 as 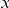 approaches infinity, the right-hand side of Equation 1 also approaches 1 as 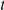 approaches infinity, for any fixed value of 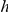. In other words, the proportion of wealth held by an arbitrarily small proportion of entrepreneurs approaches 1 through time, and essentially all the wealth of the entrepreneurs ultimately will be possessed by only a few individuals. Note that, from the right-hand side of Equation 1, the rate at which wealth concentrates is positively related to the variance in individual rates of return, 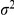, but independent of the mean rate, 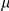. Thus, perhaps surprisingly, wealth will concentrate by this mechanism in growing, stagnant, or shrinking economies.

In this simplest model, the concentration of wealth occurs merely because some individuals are lucky by randomly receiving a series of high growth rates, and once they are ahead with exponentially growing capital, they tend to stay ahead. Because the variance in the sum of return rates is additive, over time the individuals with interest rates at the right tail of the ever-widening normal distribution come to dominate the wealth. Recall that it is the exponents that are normally distributed, not the amount of wealth, so that individuals at the high end of the distribution achieve exponentially greater fortunes. Because of the law of large numbers, our results are robust to changes in the assumption that returns on investment are drawn from a normal distribution. Annual returns drawn from any distribution that obeys the central limit theorem will give exponents whose sum approaches a normal distribution. Note that wage income, because it does not grow exponentially, is not expected to have similar wealth-concentrating effects.

**Figure 1:**
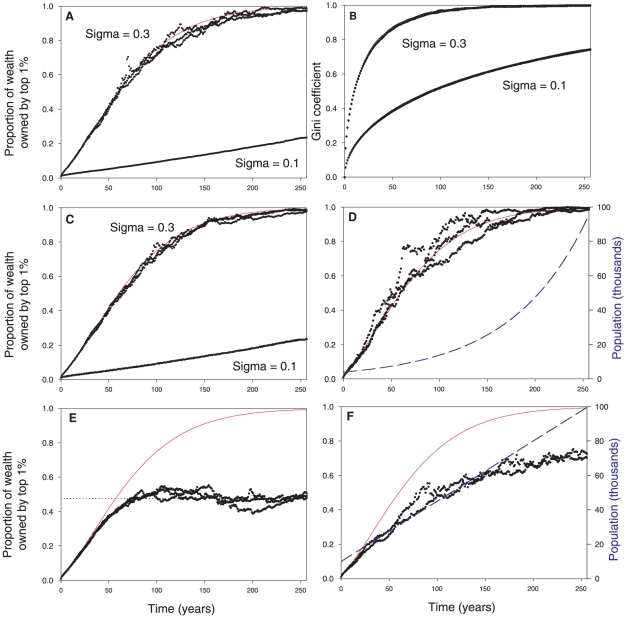
Concentration of wealth over time. All simulations start with an even distribution of wealth. Unless otherwise noted, all simulations were run with 100,000 individuals and a 5% yearly average return on investment. Red lines show the analytically expected trajectories (Eqn. 1); points show the results from individual-based simulations. Three replicate simulations were run for each high variance simulation. (A) Higher variance among individual rates of return increases the rate of wealth concentration. (B) Inequality as measured by the Gini coefficient also increases over time. (C) Wealth concentrates even when the mean growth rate varies over time, such that in some years the total economy grows and in others the economy shrinks. Average annual rates of return were randomly drawn from a normal distribution with 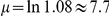
% and 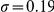, with a new value for the economy drawn each year. (D) Population growth and splitting estates among heirs does not significantly reduce rate of wealth concentration. Dashed blue line shows the growing population. (E) A tax on inherited fortunes slows and arrests the concentration of wealth. (F) Immigrants with mean wealth slow but do not arrest the concentration of wealth. Dashed blue line shows population increase from immigration.

The analytical results can be illustrated by simulations of individual-based models (Fig. 1). Although the results apply to any arbitrarily small proportion of the entrepreneurs, for presentation we track the accumulation of wealth in the top 1% of the population. The rate at which the top 1% accumulates wealth is dependent on the variance of the returns; when the variance is high, wealth concentrates quickly. For example, when the variance is 0.3, with a yearly time step it takes only 100 years for the top 1% to increase their share of the wealth from 40% (the recent level in the United States [7]) to 90% (e.g., from years 50 to 150 in Fig. 1A). These results show that, based on chance alone, some individuals will have a string of high returns and, given enough time, will accumulate the overwhelming majority of the wealth. We note that the analytical solution (1) assumes an infinite population, which guarantees the presence of some extremely lucky individuals, but our simulations show the same results in populations of only 100,000. We found similar results for simulations with populations of only 10,000 (not shown). Thus, the influence of random variation in individual returns is still clear in finite populations. The Gini coefficient also illustrates the rapid concentration of wealth that occurs in our model due to chance alone (Fig. 1B).

**Figure 2:**
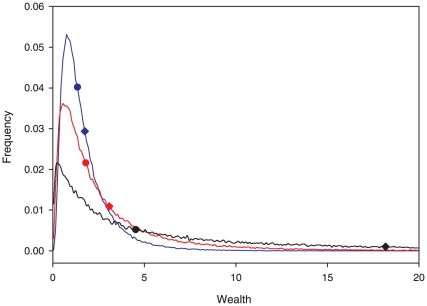
Modeled wealth distribution. In simulations with 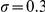, 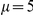
% and all 100,000 individuals starting with wealth of 1 at time zero, the lower portion of the wealth distribution is represented by the blue line at time 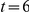, the red line at time 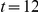, and the black line at time 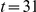. Circles indicate the median wealth and diamonds indicate the mean wealth. Note that mean wealth increases much faster than median wealth, while in this case the modal wealth, identified by the peaks of the curves, decreases. Over time, an increasing portion of the population has wealth greater than 20 units (0.01% of the population, 1%, and 18% at 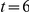, 12, and 31, respectively), which is not depicted because it falls beyond the right boundary of the graph.

The concentration of wealth has consequences for the most likely growth rate of the sum of all the entrepreneurs' capital, hereafter referred to, for brevity, as “the economy.” While the average return for an individual is 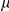, with variance 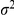, the average of all individuals' wealth 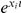, and thus the wealth of the economy as a whole, is 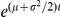. However, the mode of the distribution–the most common individual wealth—is 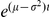. This is illustrated in Fig. 2, which shows that over time, in an economy that starts with complete equality–such that the mode, median, and mean wealth are all equal–the mean increases over time as 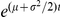, much faster than the median, which increases only as 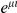, while the mode may actually decrease as 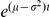. These counter-intuitive effects are direct consequences of the properties of the log-normal distribution.

Thus, the wealth of the economy as a whole grows faster than the wealth of most individuals who make up the economy. In large populations with a diverse distribution of wealth, the most likely growth rate of the economy will approach the mean of 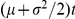, because, at any given time increment, the sum of many individual lines of capital will contribute. However, as the bulk of the wealth concentrates with a few individuals in any finite population, the growth of the economy will increasingly depend upon the returns of those few individuals and the most likely growth of the economy will decrease toward the individual modal return of 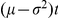. Thus, a diversity of independent lines of capital increases the most likely growth rate of the economy.

If centrally planned economies are viewed as having only one line of capital, then our results suggest that a centrally planned economy will likely have lower economic growth than an economy with diverse entrepreneurial activity. Ironically, the benefits derived from diversity in capitalist economies can be destroyed by a property inherent in the economy itself–the tendency of compounding chance, left unchecked, to concentrate wealth and effectively reduce the diversity that led to the high rates of economic growth in the first place. However, real capitalist and real centrally planned economies have many other differences that are also likely to contribute to differences in growth.

### Model robustness

The purpose of our model is to illustrate how concentration of wealth arises naturally under the simplest conditions, not to realistically describe all the features of a free market economy. However, it is important to consider whether the tendency towards concentration of wealth observed in our model is likely to be swamped by modifications that incorporate additional features of real economies. We find that our conclusions are robust to several such modifications.

(1) Real economies have periods of growth and recession, such that in some years the average rate of return is high and in other years it is low or negative. We simulated conditions in which the rate of return for the market varied normally across years with a mean annual increase of 8 percent per annum and a standard deviation of 19. These parameters reflect the distribution of real inflation-corrected returns for the S&P 500 between 1871 and 2009. Allowing for this economy-wide temporal variation in growth did not affect the concentration of wealth in our model simulations (Fig. 1C). This is consistent with our analytical prediction that wealth concentrates in growing, shrinking, and stagnant economies.

(2) Consider a model with population increase where entrepreneurs may divide an inheritance among multiple offspring. Assume that an individual dies and that his or her estate is split evenly between two offspring on average every 80 years. Individual-based simulations of these conditions show that such division of inherited wealth does not significantly affect the rate of wealth concentration (Fig. 1D).

(3) Immigration can bring new entrepreneurs to a society. Simulations show that immigrant entrepreneurs with little individual wealth speed concentration of wealth (not shown), whereas immigrant entrepreneurs with mean wealth (which is much higher than median wealth) slightly slow the rate of wealth concentration (Fig. 1F).

(4) Individuals who are relatively successful entrepreneurs today are more likely to be successful in the future, such that there is temporal autocorrelation in returns. Temporal autocorrelation acts to speed up the concentration of wealth, because individuals with initially high rates of return are likely to continue to receive high rates of return (not shown).

Thus, many of the modifications we have discussed to make the model more realistic produce an even faster rate of wealth concentration than that seen in the simplest, purely random models.

### Empirical patterns of wealth distribution

**Figure 3:**
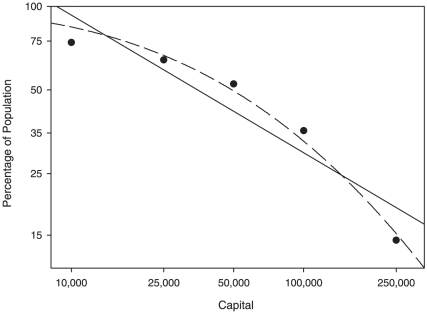
Actual distribution of U.S. wealth in 1995. Solid line represents the best fit for the Pareto distribution (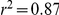) and the dashed curve represents the best fit from our model (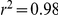). The 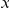-axis shows accumulated capital, log scale; the 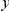-axis shows the portion of the population having that amount of capital or more, log scale. Both are two-parameter curves.

Because entrepreneurs drive patterns of wealth concentration among the richest citizens, and because one of nine Americans is self-employed, patterns of wealth concentration observed across the whole population could be predicted by our simple model. Our model predicts a log-normal distribution of wealth. In contrast, the Italian economist Pareto suggested that wealth in all societies is distributed according to what has become known as Pareto's law [20]. Much research on the distribution of wealth has been based on explaining or debating the patterns first observed by Pareto [8]. Although many studies have shown or assumed strong agreement between empirical data and Pareto's law [1], [4], [8], [21], we are not aware of any studies comparing the goodness of fit of a Pareto distribution and a log-normal distribution to modern data on the distribution of wealth. Our simple model provides a better fit than Pareto's law to data for the distribution of wealth in the United States in 1995 (i.e., as noted by May [22], these data are log-normally distributed; Fig. 3). Although much research on the distribution of wealth attempts to explain the mechanisms behind the Pareto distribution [8], our results suggest that a broader consideration of wealth distribution patterns and the mechanisms behind them is necessary. Our results are also consistent with the observations of Taleb, who argues that chance plays a large role in determining success among professional stock traders [23].

### Tax policy

Historically, wealth concentrations have varied widely. For example, in the United States, the top 1% of the population has owned between 15 and 40% of the wealth during the 20th Century [7]. The progressive income tax and the estate tax are two policies that have been used to moderate the concentration of wealth [24]–[28]. For example, the federal income tax rate for the highest income bracket was over 90% in the United States from 1951–1963 [29], effectively curtailing the concentration of wealth [24], but also creating undesirable negative incentives from high tax rates [30], [31]. Here we investigate whether a tax on inherited fortunes affecting only a small percentage of the most elite accumulated estates can effectively moderate the concentration of wealth [26].

We model an inherited fortune tax with the following assumptions: (1) life expectancy is 80 years, (2) a tax is applied only to the inherited fortunes of the wealthiest fraction of the entrepreneurs, with wealth above a designated cutoff [Fig. 1C], (3) the tax is applied by allocating a percentage of the amount of wealth greater than the cutoff and using it to benefit the whole population–for example by uniformly reducing other taxes, or by donations to causes that broadly benefit society.

The results show that an inherited fortune tax effectively halts the concentration of wealth in our models (Fig. 1E). In this example the concentration of wealth is halted by applying the tax to only a very small percentage of the population, i.e. the top 0.1%. However, the level at which the concentration of wealth is halted depends on the variance in the mean rate of return. When the variance in the rate of return is high, wealth concentrates faster and equilibrates at higher concentrations.

This analysis illustrates that limiting inter-generational transfer of wealth through an inherited fortune tax or equivalent mechanism can moderate the concentration of wealth, based on our model of entrepreneurs in industrialized societies. Recent empirical work in small-scale societies found that concentration of wealth also occurs there and is positively correlated with the degree of inter-generational wealth transmission [32], a pattern consistent with our model predictions. Jointly, both studies suggest that the degree of inter-generational wealth transmission is a factor in the concentration of wealth in a range of economic systems.

## Discussion

Empirical patterns of wealth distribution show greater concentration of wealth than is predicted by current economic models, and this wealth is disproportionately concentrated in the hands of wealthy entrepreneurs. Our analysis demonstrates that an inexorable effect of chance can lead to unlimited concentrations of wealth in the hands of a few. This occurs whenever different entrepreneurs invest in different businesses, experience different rates of return on their investments, and reinvest their capital income. Thus, inevitable random fluctuations may help explain the high concentrations of wealth that are commonly observed empirically. Indeed, the log-normal distribution of wealth predicted by our model is a better fit to recent observed wealth distribution data than is the Pareto function.

Concentrations of wealth reduce the diversity of independent capital lines that can meaningfully contribute to business growth, thus reducing the most likely aggregated business growth. Progressively deepening disparities between modal and mean wealth, as in Fig. 2, also represent increasing inequalities that may engender social instability. If society desires to promote overall economic growth and curtail the unlimited concentration of wealth, our work suggests that an inherited fortune tax–that is, an estate tax perpetually restricted to only the very largest estates (e.g. indexed to inflation)–can effectively limit the concentration of wealth under the conditions we have described. Three additional qualities of such an inherited fortune tax are worth noting: (1) the inherited fortune tax need only apply to a small percentage of the population in order to be effective [26]; (2) because it is imposed after an individual's death it maintains important economic incentives for entrepreneurs, who are able to reap the full benefits of their successful endeavors during their lifetime [26]; (3) because the concentration of wealth may reduce the most likely rate of economic growth, an inherited fortunes tax could help maintain conditions necessary for growth across the economy as a whole.
